# Practical Notes and Formulæ

**Published:** 1885-04-20

**Authors:** 


### PRACTICAL NOTES AND FORMULÆ.

**A Good Fever Mixture.**—When quinine is found to be injurious or ineffective, Dr. Giovanni recommends the following formula:

℞ Ergotine (Bonjean's),........................gr. xv
Tinct. valerianæ.............. ..............fl. ℥ ss
Aquæ .......................................fl. ℥ iij.
M. S. To be taken during the height of the fever or in the period of remittence.

Usually the good effects are obtained after one or two doses, but the remedy should be continued for some time after the subsidence of the fever. In rebellious cases the author substituted with benefit cherry-laurel water, one-half to one-drachm, for the valerian. The best results were obtained in intermittent fevers, but good effects were seen also in remittents, in the hectic of incipient phthisis, and even in puerperal fever.—*Med. and Surg. Rep.*

**Counter-Irritants.**—A writer in N. E. Med. Monthly suggests the following:

℞ Oleum tigleii..................................ʒ j

Ether sulph...................................ʒ ij

Tr. iodine.....................................ʒ v.

M. S.

This excellent counter-irritant is applicable where it is not necessary to produce too much effect. It is particularly nice for children.

The other prescription is stronger and is intended for cases where the doctor wants a more decided effect:

℞ Oleum tigleii................................ʒ ij

Ether fort....................................ʒ vj

Tr. iodine.................................... ʒ ij

Pot. iodide...................................℈ j

Iodine resub.................................grs. x.

M. S. Counter-Irritant Fort.—*Theodore Ellwood, M. D.*

**Improved Formula for Syrup of Dover's Powder.**—I enclose a formula from which I have several times made lots of a gallon at a time, and find that it will keep indefinitely. We have some now in stock that has been made by it for at least six months, and it is apparently as fresh as when just made.

Syrup Dover's Powder—

℞ Sulph. potash......................... 1 oz. 32 grs.
Hot water............................ 1 pint.
Sugar...............................12 ounces.
Tinct. opium (1870).................... 7 drachms.
Fl. ext. ipecac.........................32 minims.

Dissolve sugar and sulph. potash in the hot water, and evaporate to 15 fl. ounces. Strain, and when cool add tinct. opii and fl. ext. ipecac.

This makes a syrup representing 5 grains Dover's Powder to the fl. drachm.—*Dr. Shaffer in Drug. Cir.*

**Chronic Ague.**—

℞ Tincture of Cinchonæ Co., }

Tincture of Capsicum, } aa..........℥ j

Elixir vitriol..........ʒ ss

Spt's of Lavender Co..........℥ j

Essence of Wintergreen..........℥ ss

Quinæ sulph..........ʒ j.

Mix. Sig. Dose, a teaspoonfull three or four times a day.—*A. N. Cruther, M. D., in New. Eng. Med. Monthly*

**Nurse's Sore Mouth.**—A writer in American Medical Journal suggests the following as an excellent remedy in this affection:

℞ Mother tincture eupatorium aromaticum..........ʒ ij

Fluid hydrastis..........ʒ ij

Water..........℥ iijss.

M. S. One teaspoonful every hour.

**Pruritus Vulvæ.**—

℞ Sodii hyposulphitis..........℥ iv

Glycerini..........ʒ ij

Aquæ destilat..........ad. ℥ vj.

M. Sig. As lotion.—Fox.

This simple combination has proved very effective in that troublesome and very annoying malady, pruritus vulvæ, and also in tinea versicolor.—*N. E. Med. Monthly.*

**Hypodermic Injection of Apomorphine.**—

℞ Hydrochlorate of apomorphine..........grs. ij
Water..........M. 100.
Dissolve and filter.—*Nat. Drug.*

**Extemporaneous Solution of Hydrobromate of Quinine.**—

℞ Bromide of potassium..........grs. 162
Tartaric acid..........grs. 198
Sulphate of quinine..........grs. 60
Water..........fl. ℥ 3.

Mix and filter.—*Nat. Drug.*

**Glycerite of Alum.**—Dissolve 1 ounce of powdered alum in 5 ounces glycerin by the aid of gentle heat.

This is recommended by Mr. R. W. Parker as a local astringent as powerful as tannin, but less disagreeable and quite compatible with iron preparations.—*Brit. Med. Jour.*

